# Nanoliposomes as a Therapeutic Tool for Alzheimer’s Disease

**DOI:** 10.3389/fnsyn.2020.00020

**Published:** 2020-05-25

**Authors:** Lara Ordóñez-Gutiérrez, Francisco Wandosell

**Affiliations:** ^1^Department of Molecular Neurobiology, Centro de Biología Molecular “Severo Ochoa” (CSIC-UAM), Universidad Autónoma Madrid, Madrid, Spain; ^2^Centro de Investigación Biomédica en Red de Enfermedades Neurodegenerativas (CIBERNED), Madrid, Spain

**Keywords:** neurodegeneration, Alzheimer therapy, immunotherapy, nanoparticle, liposomes, immunoliposomes

## Abstract

The accumulation of extracellular amyloid-beta (Aβ), denoted as senile plaques, and intracellular neurofibrillary tangles (formed by hyperphosphorylated Tau protein) in the brain are two major neuropathological hallmarks of Alzheimer’s disease (AD). The current and most accepted hypothesis proposes that the oligomerization of Aβ peptides triggers the polymerization and accumulation of amyloid, which leads to the senile plaques. Several strategies have been reported to target Aβ oligomerization/polymerization. Since it is thought that Aβ levels in the brain and peripheral blood maintain equilibrium, it has been hypothesized that enhancing peripheral clearance (by shifting this equilibrium towards the blood) might reduce Aβ levels in the brain, known as the sink effect. This process has been reported to be effective, showing a reduction in Aβ burden in the brain as a consequence of the peripheral reduction of Aβ levels. Nanoparticles (NPs) may have difficulty crossing the blood-brain barrier (BBB), initially due to their size. It is not clear whether particles in the range of 50–100 nm should be able to cross the BBB without being specifically modified for it. Despite the size limitation of crossing the BBB, several NP derivatives may be proposed as therapeutic tools. The purpose of this review is to summarize some therapeutic approaches based on nanoliposomes using two complementary examples: First, unilamellar nanoliposomes containing Aβ generic ligands, such as sphingolipids, gangliosides or curcumin, or some sphingolipid bound to the binding domain of ApoE; and second, nanoliposomes containing monoclonal antibodies against Aβ. Following similar rationale NPs of poly(lactide-co-glycolide)-poly (ethylene glycol) conjugated with curcumin-derivate (PLGA-PEG-B6/Cur) were reported to improve the spatial learning and memory capability of APP/PS1 mice, compared with native curcumin treatment. Also, some new nanostructures such as exosomes have been proposed as a putative therapeutic and prevention strategies of AD. Although the unquestionable interest of this issue is beyond the scope of this review article. The potential mechanisms and significance of nanoliposome therapies for AD, which are still are in clinical trials, will be discussed.

## Introduction

Currently, the treatment of Alzheimer’s disease (AD) pathology is one of the most disappointing examples of exploration for new drugs in Biomedicine. Despite a great number of putative molecular targets described in the literature and significant positive data from animal models, there are only a few symptomatic treatments offered, with no cure yet available (Mangialasche et al., 2010; Schneider et al., 2014). The reasons for this can be attributed to numerous factors: (1) the lack of selectivity and specificity of anti-AD drugs; (2) the inability of most drugs to cross the blood-brain barrier (BBB); (3) the selection of only one target to test efficacy, since AD has a multifactorial and complex etiopathology; or (4) the selection of patients in an advanced state of pathology. The recruitment of patients in very early stages of the disease was initially complex due to the lack of good, early diagnostic markers. This issue is now address using a combination of CSF biomarkers, blood biomarkers, MRI, Amyloid PET, Tau PET, et cetera (Bateman et al., 2012). These combinatory and additional approaches are being used to define common or differentiating biological denominators across the different neurodegenerative diseases to define stages of pathophysiological progression, characterize systems-based intermediate endophenotypes, and validate multi-modal novel diagnostic systems biomarkers. All these data will favor more robust clinical intervention trial designs (Hampel et al., 2018).

AD is the most common neurodegenerative disorder, and it is characterized by the presence of two pathological hallmarks in the brain: the deposition of extracellular amyloid plaques (senile plaques); and the formation of intracellular neurofibrillary tangles (Alzheimer et al., 1995). Amyloid plaques mainly contain the amyloid-beta (Aβ) peptide, which is released by proteolytic cleavage of the amyloid precursor protein (APP; Selkoe, 2001; Hardy and Selkoe, 2002; Selkoe and Hardy, 2016). Indeed, Aβ peptides are generated by two consecutive proteases, β-secretase and γ-secretase, which leads to the production of different amyloid varieties such as Aβ40, Aβ42 or Aβ43 (Iwatsubo et al., 1994; Haass and Selkoe, 2007; Olsson et al., 2014). In contrast, the intracellular neurofibrillary tangles are composed of the hyper-phosphorylated and polymerized Tau protein (for review see i.e., Hernandez and Avila, 2008). In addition to these histopathological hallmarks, a series of cellular events occur throughout the progression of the pathology in the brain parenchyma: microglia and astrocyte activation, synaptic dysfunction, axonal transport failure, and even neuronal death.

The most accepted hypothesis proposes that oligomerization of Aβ peptides trigger the polymerization and accumulation of amyloid, which generates the senile plaques. However many data strongly suggested that the oligomeric forms of Aβ are probably the most toxic species. And several hypothesis support this toxic effect, such as the capacity to Aβ and Aβ-peptides to bind membrane components (Barrett et al., 2012), increasing dysfunction in ER and/or ROS and mitochondrial homeostasis, et cetera (Cheignon et al., 2018; Poirier et al., 2019; Ashkavand et al., 2020).

In parallel, several characterized neuronal dysfunctions trigger the hyper-phosphorylation and accumulation of Tau (Hardy and Selkoe, 2002).

Numerous strategies have been developed to target amyloid generation or accumulation, as well as Tau accumulation or hyper-phosphorylation. Also, many different compounds such as calcium or ROS modulators, glutamate receptor antagonists, cholinergic transmission modulators, cholinesterase inhibitors, some kinase inhibitors, etc., have been assayed in preclinical and clinical trials (for a review see i.e., Mangialasche et al., 2010; Schneider et al., 2014). Unfortunately, many candidate drugs have failed to show a clinical benefit in established, early, or prodromal disease, or in those with high AD risk, and the few symptomatic treatments are limited to the targeting of cholinergic deficits and glutamatergic dysfunction (Tayeb et al., 2012). Thus immunotherapy is or could be one complementary method that has been proposed for its ability to reduce the accumulation of Aβ and potentially treat the underlying cause of AD.

## How to Find New Therapeutic Alternatives Against Alzheimer’s Disease

Strategies have been reported using several compounds against polymerization, as well as antibodies against different regions (Mangialasche et al., 2010) or different oligomerization states of the Aβ peptide (Zhao et al., 2017). One such strategy is based on the hypothesis that there exists an equilibrium between the Aβ levels in the brain and the peripheral blood. Thus, it was postulated that if this equilibrium were altered by enhancing peripheral clearance, it would reduce Aβ levels in the brain. Through this so-called “sink effect,” it was shown that peripheral reduction of Aβ levels provokes a reduction in Aβ brain burden (Matsuoka et al., 2003; Biscaro et al., 2009). This initial hypothesis was further supported by the fact that immunotherapy facilitates clearance of Aβ in animal models of AD (DeMattos et al., 2001; Lemere et al., 2003; Sutcliffe et al., 2011). However the hypothesis is still a matter of discussion, while some data did not support this theory, for instance, some peripheral depletion of Aβ does not affect central levels of Aβ (Henderson et al., 2014). Even in our experiments the same preparation of inmunoliposomes, had no brain effect in the AD model, with adult mice, but it did with older mice (Ordóñez-Gutiérrez et al., 2017). Although in the case of aged mice, the nanoliposomes were more effective than the same amount of free IgG. This supports, at least in part, a hypothesis of peripheral effect more, or more powerful than the central one, which we cannot rule out.

Certainly, the mechanisms by which antibodies against Aβ can clear brain Aβ remains to be clarified. Translocation of antibodies across the BBB, if it is even possible, is still a controversial issue. One of the main issues is whether the amount of antibodies crossing the BBB is sufficient to be therapeutically relevant. One possibility is that IgG translocation is modulated by the BBB at different disease stages; perhaps in some initial stages of AD, the BBB is compromised to permit IgG’s translocation. It is essential to address the ability of the immune system to clear the antibody-amyloid complexes without provoking excessive neuroinflammation.

However, the sink effect hypothesis proposes that a reduction of Aβ in the peripheral plasma generates a concentration gradient across the BBB, which promotes efflux of brain Aβ into the blood (DeMattos et al., 2001; Lemere et al., 2003). Alternatively, other groups have proposed that a “significant Aβ pool” may have been generated in the periphery and then transfers to the brain through the BBB. Consequently, any strategy that reduces plasma Aβ levels could effectively decrease Aβ transportation and deposition in the brain, thereby minimizing plaque formation (Sutcliffe et al., 2011).

The first approach for immunotherapy was an *in vitro* assay that revealed that anti-Aβ antibodies greatly reduced fibrillary formation, disrupting pre-formed fibrils, and preventing neurotoxicity (Solomon et al., 1997). The initial data reported that full-length, aggregated Aβ42 with Freund’s adjuvant could reduce plaque load *in vivo*, in an AD mouse model with no obvious toxicity (Schenk et al., 1999). Similarly, later studies using Aβ42 or Aβ homologous peptides with different adjuvants not only strongly reduced Aβ plaques but also prevented cognitive deficits (Janus, 2003; Lemere et al., 2006; Morgan, 2006, 2011; Sigurdsson, 2008). These data suggest that an immune response is generated in the mouse against the amyloid peptide Aβ42 that must be responsible for the therapeutic effect, whether the antibodies cross the BBB or not. However, it is important to note that the results obtained in mouse models are hardly reproducible in humans. Not in vain, to reproduce AD hallmarks in mouse models, it is necessary to express two (APP and Tau) or three (APP, Presenilin, and Tau) dominant mutations. And we have to remind that Tau mutations are not FAD mutations, but only found in FTD and other tauopathies, thus the mice model of triple mutations is genetically speaking, not a good AD model.

These experiments opened up the second strategy: peripheral injections of anti-Aβ monoclonal antibodies (MAbs). Interestingly, peripheral injections of MAbs also reduced Aβ plaque burden and behavior, with no evidence of toxicity in the immunized mice (Bard et al., 2000; DeMattos et al., 2001; Lemere, 2013; Wisniewski and Goñi, 2015). All these data strongly support the hypothesis that the therapeutic effect of the vaccine was likely mediated by the humoral response (Wisniewski and Goñi, 2014). The data from preclinical studies served to launch some phase I clinical trials (Bayer et al., 2005; Wisniewski and Frangione, 2005), wherein full-length Aβ42 and different adjuvants were assayed. More than 50% of the healthy subjects generated an anti-Aβ humoral response, in some cases with different Th-1/Th-2 lymphocyte responses (Pride et al., 2008). A complementary phase II trial was initiated in 2001, however, 6% of the immunized patients presented symptoms of aseptic meningoencephalitis, and the trial was terminated early in 2002 (Wisniewski and Frangione, 2005; Boche et al., 2010).

At present, several passive immunization trials are underway at either Phase I, II, or III, based on different fragments of Aβ1–42 and/or different formulations^1^. However, several phase III trials (such as Bapineuzumab and Solanezumab) failed to show overall clinical improvement or any clear disease-modifying results (Doody et al., 2014; Salloway et al., 2014a,b; Panza et al., 2019). Thus, immunotherapeutic approaches have thus far generated mixed therapeutic outcomes, more positive in animal AD models than in patients (for a more recent update see: Panza et al., 2019). These discrepancies may be due to species differences and considerations in age, disease stage and associated variations in BBB permeability, as well as adverse neuroinflammatory effects (Bard et al., 2000; DeMattos et al., 2001; Demattos et al., 2012; Orgogozo et al., 2003; Doody et al., 2014; Salloway et al., 2014a,b).

At present only a few immunotherapies in clinical trials II and III are still in progress (i.e., Aducanumab—BIIB037, or BAN2401; Logovinsky et al., 2016; Sevigny et al., 2016; Arndt et al., 2018). In the case of Aducanumab after two initial failed analyses of the phase 3 AD trials, in 2019 they reanalyzed data the company showed some significant findings and a subset from the second trial supports these positive findings. In Oct 2019, the company did apply for the US Food and Drug Administration (FDA) marketing approval of Aducanumab.

After these initial unsuccessful attempts, different approaches have since been initiated, some of which are based on new antibodies targeting different regions of Aβ (Boutajangout et al., 2019; Zhang et al., 2019), Tau (Sigurdsson, 2008, 2018) or BACE (Atwal et al., 2011), or stem from new formulations of antibody presentation (Hasegawa et al., 2010; Wong et al., 2019).

## Nanoparticles as A New Tool Against Neurodegenerative Diseases

NPs are materials or devices on a scale between 1–100 nm, and represent an innovative and promising approach, mostly due to their physicochemical features and the possibility of multi-functionalization, allowing them to confer more than one feature at the same time, such as the ability to cross the BBB. Recently, nanomaterials have emerged as an alternative to deliver different drugs for several pathologies including cancer and brain diseases.

**Figure 1:**
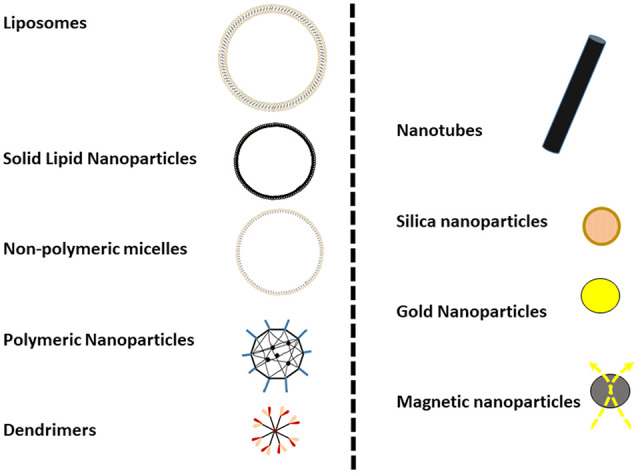
Graphical representation of some commonly used nanoparticles (NPs). NPs are typically not more than 100 nm in diameter (as in the cases of liposomes) and some metallic nanoparticles, such as gold or magnetic NPs, could be less than 10 nm.

At present, several classes of NPs (i.e., solid lipid nanoparticles (SLN), PLA/PLGA NPs, dendrimers, nanofibers, nanotubes, nanoliposomes, nanogels) are available for preclinical or biomedical use with different physicochemical features and applications (see some schematic representation in Figure 1).

In the field of Nanomedicine, the “pros and cons” of this variety of NPs are still being defined. One of the common advantages of the NP is the possibility of multifunctionalization, coupled to their ability to carry drug cargos, included BBB-impermeant drugs. In particular, for brain drug delivery is that proper surface multifunctionalization may promote at the same time either their targeting of the BBB or the enhancement of its crossing. This would be the case of, for instance, Liposomes, SLN, Polymeric, and Non-polymeric NPs. All these would have some surface multifunctionalization and carrying drug cargo (hydrophilic and hydrophobic pharmaceuticals). In contrast, the size or the size after multifunctionalization would be the negative aspect.

Some of them have been used in preclinical models for neurodegenerative diseases, however specific nanoliposomes, such as Doxorubicin Liposomal (Doxil^®^) are available for cancer therapy, alone (Su et al., 2015; Gong et al., 2016; Shi et al., 2017) or in combination (i.e: Monk et al., 2020).

A second group, in which they would be Nanotubes or Silica, Gold, Iron (magnetic or not) NPs, have in its favor the production in small sizes, their high chemical, and biological stability or some specific properties, such the capacity to be heated (magnetic particles; Wu et al., 2019). These NPs have the advantage of being very small and homogeneous but of greater difficulty to modify and derivatize their surface. However, most of these NPs group have not been derivatized or not used in Neurodegeneration models, so far. Only magnetic iron NPs are having a broader development in cancer treatments (Wu et al., 2019).

Moreover, many NPs can be functionalized by covalent conjugation to various ligands (such as antibodies, proteins, or aptamers) to target specific tissues. Another important consideration is the biocompatibility, in the group of Liposomes, SLN, Polymeric, and Non-polymeric NPs and dendrimers, many of these backbones could be easily metabolized, with low toxicity. However, in the case of metal derived NPs a more exhaustive pharmacokinetic analysis has to be done.

In the field of neurodegeneration, NPs are an interesting biomedical tool with the potential to solve different problems. At a therapeutic level, they offer the possibility of being multi-functionalized; for example, one ligand may enable the crossing of the BBB to deliver a second ligand, the drug. They may also have uses in the diagnostic field, where one ligand may enhance the capacity to bind amyloid plaques, whereas the second would facilitate “molecular imaging” techniques (Mori et al., 2012; Bao et al., 2013; Liu et al., 2016).

At present, a plethora of different NPs have been assayed in cellular and animal models, and in many cases are already in the initial stages of a preclinical study in models of AD (i.e., Ordóñez-Gutiérrez et al., 2015, 2017; Sanchez-Lopez et al., 2017; Carradori et al., 2018). The field of nanotechnology devoted to therapeutic purpose is growing exponentially, with many broad or more specialized reviews having been published recently (i.e., Karthivashan et al., 2018; Aliev et al., 2019; Formicola et al., 2019).

Some of these NPs have had very little development, for example, micellar nanocarriers, and only some of them have been tested in AD models (Singh et al., 2018). In contrast, more data is available on the use of Dendrimers as a class of well-defined branched polymers that are chemically synthesized with a well-defined shape, a size that could be used not only for site-specific medications but for MRI diagnosis and gene therapy. For example, some initial reports indicated that gallic acid-triethylene glycol (GATA) dendrimers reduced amyloid toxicity (Klajnert et al., 2012). More recently polyamidoamine (PAMAM) and polypropyleneimine (PPI) dendrimers nanomacromolecules have received more attention as candidates for the treatment of neurodegenerative diseases (Aliev et al., 2019).

Nevertheless, in this review, we will focus only on liposome-based therapy.

## Liposome-Based Therapy Against Aβ Plaques

Liposomes are a biocompatible, highly flexible drug delivery system, with the potential to carry many different types of bioactive molecules, both on the inside and/or outside of the particle. Their biochemical composition and formulation facilitate numerous modifications. We can consider two options: First, a non-targeted liposome, which can transport the compound directly; and second, “targeted” liposomes which are designed to interact with specific molecular targets relevant to the diagnosis, treatment, or prevention of AD. Regardless, liposomes can incorporate hydrophilic (entrapped in the aqueous core) or hydrophobic compounds (contained within the lipid bilayer). Indeed, incorporation of multiple compounds can grant therapeutic activity while also facilitating the passage of the BBB (Mancini et al., 2016, 2017; Vieira and Gamarra, 2016; Dal Magro et al., 2017), as recently reported using two ligands: one external ligand to favor BBB entry and another with therapeutic potential (Balducci et al., 2014).

## Liposome Ligands Against Aβ: Compounds/Drugs Vs. Antibodies

*In vivo* assays using AD animal models have been used to follow different strategies, either using synthetic or natural compounds with previously reported affinity for Aβ peptides, such as curcumin-derivatives (Thioflavin-T), anionic phospholipids (gangliosides), or antibodies against specific Aβ regions. In many cases, we have direct information about how these compounds may affect AD hallmarks. However, there is not much comparative data regarding whether some of these compounds are more effective when administered alone or in a NP-bound form. In the next section, we will summarize some of these comparative data.

### Molecules That Might Bind Aβ

Curcumin is a naturally occurring phytochemical phenol and is a potent antioxidant and anti-inflammatory compound. It is known that curcumin targets Aβ, interferes with amyloid polymerization, amyloid plaque formation, and amyloid toxicity directly (Kim et al., 2001; Yang et al., 2005), and indirectly enhance Aβ clearance (Zhang et al., 2006; Begum et al., 2008), suggesting a potential role for prevention or treatment of AD. Curcumin derivatives have been used including curcumin-decorated nanoliposomes, as liposomes exposing the curcumin derivative have an extremely high affinity for Aβ42 fibrils (Mourtas et al., 2011). More recently, NPs of poly(lactide-co-glycolide)-poly (ethylene glycol) conjugated with curcumin-derivate (PLGA-PEG-B6/Cur) were reported to improve the spatial learning and memory capability of APP/PS1 mice, compared with native curcumin treatment. This report indicated that PLGA-PEG-B6/Cur could reduce hippocampal Aβ formation/deposit and Tau hyperphosphorylation. Thus, they suggested that NPs conjugated with curcumin derivatives could hold promise as a drug for the treatment of AD (Fan et al., 2018).

Similarly, several groups (including ours) have demonstrated that liposomes containing phosphatidic acid (PA) and cardiolipin (CL) reduce Aβ levels in APP/PS1 transgenic mice. This data came from a European consortium, part of the Seventh Framework Programme (FP7/2007-2013; NAD: NPs for Therapy and Diagnosis of Alzheimer Disease^2^), devoted to the analysis of NPs to treat AD. The initial data showed that functionalized liposomes with PA and CL still maintain the ability to bind Aβ42 (Balducci et al., 2010). Then, we tested whether intraperitoneal injection of small unilamellar liposomes containing either PA or CL could reduce the amyloid burden in APP/PS1 transgenic mice. We observed that this treatment significantly reduced the amount of Aβ in the plasma, with only a tendency to decrease Aβ levels in the brain. Nevertheless, this dosing regimen did modulate Tau phosphorylation and glycogen synthase kinase 3 activities in the brain, suggesting that the targeting of circulating Aβ may be therapeutically relevant in AD. In contrast, treatment with plain liposomes was devoid of any effect (Ordóñez-Gutiérrez et al., 2015).

We initially considered that these unilamellar liposomes were not able to cross the BBB even though we detected neuronal changes in the AD mouse model, suggesting some biochemical connection between the putative effect on the periphery with changes inside the central nervous system (CNS), as inferred from the modification in the phosphorylation levels of neuronal-specific proteins (Ordóñez-Gutiérrez et al., 2015). Thus, the next approach was to test bifunctionalized liposomes (mApoE–PA-Lipo), with ApoE-peptides to improve BBB passage (Arsenault et al., 2011). This report indicated that the uptake of nanoliposomes by cell monolayers was enhanced by containing acidic phospholipids with the ApoE-peptide-functionalization, and was higher with the 141–150aa fragment than with its tandem dimer. Intraperitoneal injection of mApoE–PA–Lipo for 3 weeks (three injections per week) showed a decrease in brain-insoluble Aβ1–42 and in the area occupied by plaques, as detected histologically. Plaque reduction was confirmed in APP23 transgenic mice (15 months-of-age) either histologically or by PET imaging with [^11^C] Pittsburgh compound B (PIB). Also, the novel object recognition test showed that the treatment ameliorated the mice’s impaired memory.

These data suggest that bifunctionalized liposomes destabilize brain Aβ aggregates and promote peptide removal across the BBB and its peripheral clearance (Balducci et al., 2014; Bana et al., 2014). All these data strongly support the idea that a similar multi-functionalized therapeutic device can be considered as a candidate for the treatment of AD (Formicola et al., 2019).

### Antibodies Against Aβ

All these data open the next question: can a high-affinity ligand, such as an antibody, be an optimal therapeutic option after binding to NPs? As indicated above, different strategies for treatment and prevention of AD are currently under investigation, including passive immunization with anti-Aβ MAbs. Even though few of them remain in clinical trials (Panza et al., 2014, 2019), some have been assayed using different adjuvants, suggesting that the final *in vivo* inoculation may be essential for a therapeutic effect, even though it is generally accepted that a better set of biomarkers is essential to recruit and to follow patients in clinical trials.

However, the manipulation of specifically engineered nanomedicines to cross the BBB and target the selected “site of action” (i.e., Aβ, Tau, glial activation, inflammatory response, etc.), is one of the most interesting innovations in drug delivery. This could represent a promising choice for treatment or even early diagnosis of AD (Dal Magro et al., 2017; Song et al., 2018). In many cases, we did not have a comparative analysis showing whether a specific MAb used in AD models is more effective alone or in NP-bound form. Thus, a comparative analysis was performed to determine whether a new specific MAb against Aβ1–42 would be more effective when free or when bound to nanoliposomes.

The MAb was generated in mice by immunization with Aβ and after purification and characterization, a high affinity for both Aβ monomers and fibrils (0.08 and 0.13 nm, respectively) was confirmed. After biotinylation and binding to the liposome, the affinity was lower, although still in the low nanomolar range (2.1 and 1.6 nM for Aβ monomers and fibrils, respectively). Control IgG-decorated liposomes were generated by the same methodology (Canovi et al., 2011). Interestingly, only the Aβ-MAb-liposomes markedly bound to Aβ monomers and fibrils, and in this conformation, the affinity was determined around 0.5 and 2 nM (for liposomes with high and low Aβ-MAb density, respectively). The ability of Aβ-MAb-liposomes to bind to Aβ fibrils was additionally confirmed by an ultracentrifugation technique, in which interactions occur in solution under physiological conditions (Canovi et al., 2011). This type of Aβ-MAb-liposome may be additionally modified without a major reduction of its affinity (Markoutsa et al., 2012), confirming the potential of these NPs for the diagnosis and therapy of AD.

**Figure 2:**
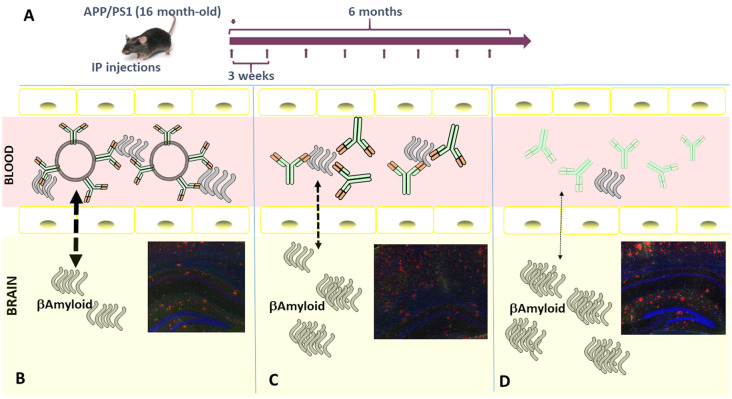
**(A)** Schematic representation of the *in vivo* injection protocol used. Imnunoliposomes containing MAb against Aβ peptide** (B)**, or the same MAb alone **(C)**, or control IgG **(D)** were injected following the procedure indicated. Schematic representation of how MAb against Aβ peptide may imbalance the Aβ peptide equilibrium from the brain to blood through blood-brain barrier (BBB). Our data indicated that Lip-MAb reduces the presence of amyloid inside the brain better than the same MAb alone, or irrelevant mouse IgG. Immunofluorescence analysis of mouse brain sections. Brain coronal fixed sections from mice treated with and Lip-MAb **(B)**, MAb **(C)**, or with non-specific IgG **(D)**, were stained with 6E10 antibody (red) and an anti-glial fibrillary acidic protein (GFAP) antibody (green). Note that these images correspond with representative sections similar to those contained in the manuscript (Ordóñez-Gutiérrez et al., 2017).

Using a similar batch of Aβ-MAb-pegylated-liposome, we have performed two trials in different aged mice (adult and old), injecting APP/PS1 transgenic mice intraperitoneally. Our rationale was that in an “old mouse” AD model, perhaps the BBB is compromised and the percentage of nanoliposomes able to cross could increase. Thus, first, we tested in 10-month-old (adult) mice divided into four treatment groups (Aβ-MAb-liposome, plain-liposome, Aβ-MAb alone, and control–IgG1 MAb) and intraperitoneally injected for 4 months. In the second assay, 16-month-old (elderly) mice were divided into three treatment groups (Aβ-MAb-liposome, Aβ-MAb, and control–IgG1 MAb) and treated for 6 months by the same route of administration. In all cases, 4 mg of liposomes and 150μg of antibody were given every 3 weeks (Ordóñez-Gutiérrez et al., 2017). This version of immunoliposomes dramatically reduced circulating and brain levels of Aß1–40, and particularly Aß1–42, in “elderly” but not “adult” APP/PS1 transgenic mice upon repeated intraperitoneal administration. A detailed analysis of the treated brains showed that the immunePEG liposome-mediated reduction in amyloidosis correlated with lower levels of glial fibrillary acidic protein (GFAP) and reactive glia (GFAP-positive cells). This treatment also lowered the ratio of phosphorylated Tau to total Tau. Thus, the therapeutic efficacy of immunoliposome treatment was age-dependent and superior to free MAb administration (at an equivalent antibody dose; Ordóñez-Gutiérrez et al., 2017; see the schematic representation in Figure 2).

In summary, as a proof-of-concept, the use of NPs conjugated with one or more ligands opens a new field of therapeutic approaches to neurodegenerative diseases, for several reasons. They can be multi-functionalized, able to cross BBB and they can be more effective than a similar amount of drug alone.

## Tau Targeting and Other Related Therapies

As the second important hallmark of AD, it is important to develop specific therapies directed at Tau. Although the pathophysiology of Tau-mediated neurodegeneration is not completely clear, Tau hyper-phosphorylation, oligomerization, and polymerization have been proposed as the likely pathological processes causing neurodegeneration (Yoshiyama et al., 2013). Thus, different therapeutic approaches have been proposed, some of which have been deeply studied in several assays (Novak et al., 2018a).

In initial Tau immunotherapy programs, it was reported that various active and passive Tau immunizations diminished pathology and improved brain functions (including cognition) in different mouse models. Both extra- and intracellular pathways were likely involved (Sigurdsson, 2014). It was proposed that some antibodies may block the spread of Tau pathology *via* microglial phagocytosis of the antibody-Tau complex and facilitate lysosomal Tau clearance in neurons after endosomal uptake (Sigurdsson, 2016, 2018).

Indeed, some years later, an active immunotherapy Tau-based strategy reached Phase II. This Tau peptide vaccine produced an important reduction in the levels of hyper-phosphorylated Tau and neurofibrils by approximately 95% (Kontsekova et al., 2014). An adapted liposome-based amyloid vaccine was incorporated, using a synthetic phosphorylated peptide to mimic a phosphor-epitope of Tau protein. Long-term vaccination improved symptoms and reduced Tauophaty in P301L mice and this project is in Phase I (Theunis et al., 2013). And more recently AADvac1 tau vaccination trial reached Phase II (Novak et al., 2018b).

Alternatively, some authors have proposed a different approach to inhibit Aβ production *via* antibodies against β-secretase (BACE), as Aβ is produced in a two-step proteolytic process of APP, initiated by BACE1 and followed by γ-secretase. Due to its apparent rate-limiting function, BACE1 appears to be a prime target to prevent Aβ generation in AD.

Two different approaches have been reported, using antibodies against the β-secretase cleavage site of the amyloid precursor protein. Some data indicates that these antibodies reduce endogenous BACE1 activity and Aβ production in human cell lines expressing APP and in cultured primary neurons. And more importantly, long-term systemic administration of anti-APP beta-site antibodies to Tg2576 transgenic mice improved mouse cognitive functions associated with a reduction in both brain inflammation and the incidence of microhemorrhage (Rakover et al., 2007). The second alternative involves the direct targeting of BACE 1 since systemic dosing of mice and nonhuman primates with anti-BACE1 resulted in sustained reductions in peripheral Aβ peptide concentrations. Anti-BACE1 has been reported to be highly selective and does not inhibit the related enzymes BACE2 or cathepsin D. Thus, BACE1 can be targeted in a highly selective manner through passive immunization with anti-BACE1, providing another potential approach for treating AD (Atwal et al., 2011). Thus, therapeutic success with anti-BACE1 may depend on improving antibody uptake into the brain or being multi-functionalized in NPs.

The field of nanotechnology applied to neurodegeneration is expanding rapidly, and new materials and applications should be extensively analyzed. For example, some studies suggest that neuron-derived exosomes may participate in Aβ clearance in the brains. These authors described that neuronal exosomes, a subtype of extracellular nanovesicles, enwrap, or trap Aß and transport it into microglia for degradation. They support the hypothesis that the pathway of Aß clearance by the exosomes may have potential significance as a novel therapeutic and prevention strategies of AD. As a new nanotherapy, it should be carefully analyzed (Yuyama and Igarashi, 2017). Although of unquestionable interest, this issue is beyond the scope of this review.

## Conclusions

The search for effective therapies against AD is at an important crossroads. A significant number of compounds with different targets have been tested and discarded. A series of challenges loom, foremost among them being the selection of patients; it is essential to find a group of molecular markers that facilitate diagnosis of pathology in prodromal phases. A set of markers that correctly distinguish Mild Cognitive Impairment (MCI) from pre-AD is needed for clinical trials, and given that this pathology can start 15–20 years before symptoms appear, this is a monumental task.

Secondly, new therapeutic targets must be found or reformulated to ensure the passage of the BBB. For this goal, the use of NPs seems quite promising, although more work needs to be done in animal models to confirm the improvement of this multifunctional formulation over the administration of the compound alone.

## Author Contributions

LO-G is the supervisor of our data and collaborations from the APP/PS1 mice model in our group. LO-G and FW accumulated the data and wrote the present review.

## Conflict of Interest

The authors declare that the research was conducted in the absence of any commercial or financial relationships that could be construed as a potential conflict of interest.

## Footnotes

^1^http://www.clinicaltrials.gov

^2^http://www.nadproject.eu/
